# Longitudinal, Interdisciplinary Home Visits Versus Usual Care for Homebound People With Advanced Parkinson Disease: Protocol for a Controlled Trial

**DOI:** 10.2196/31690

**Published:** 2021-09-14

**Authors:** Jori E Fleisher, Serena Hess, Brianna J Sennott, Erica Myrick, Ellen Klostermann Wallace, Jeanette Lee, Maya Sanghvi, Katheryn Woo, Bichun Ouyang, Jayne R Wilkinson, James Beck, Tricia J Johnson, Deborah A Hall, Joshua Chodosh

**Affiliations:** 1 Section of Movement Disorders Department of Neurological Sciences Rush University Medical Center Chicago, IL United States; 2 Western Michigan University Homer Stryker MD School of Medicine Kalamazoo, MI United States; 3 Social Work and Community Health Rush University Medical Center Chicago, IL United States; 4 Yale College Yale University New Haven, CT United States; 5 Department of Neurological Sciences Rush University Medical Center Chicago, IL United States; 6 Corporal Michael J Crescenz VA Medical Center Philadelphia, PA United States; 7 Department of Neurology University of Pennsylvania Philadelphia, PA United States; 8 Parkinson's Foundation New York, NY United States; 9 Department of Health Systems Management Rush University Medical Center Chicago, IL United States; 10 Division of Geriatric Medicine and Palliative Care Department of Medicine New York University Grossman School of Medicine New York, NY United States; 11 Department of Veterans Affairs New York Harbor Healthcare System New York, NY United States

**Keywords:** home visits, telehealth, Parkinson disease, homebound, palliative care, quality of life, interdisciplinary care, caregiver, caregiver strain

## Abstract

**Background:**

The current understanding of advanced Parkinson disease (PD) and its treatment is largely based on data from outpatient visits. The most advanced and disabled individuals with PD are disconnected from both care and research. A previous pilot study among older, multimorbid patients with advanced PD demonstrated the feasibility of interdisciplinary home visits to reach the target population, improve care quality, and potentially avoid institutionalization.

**Objective:**

The aim of this study protocol is to investigate whether interdisciplinary home visits can prevent a decline in quality of life of patients with PD and prevent worsening of caregiver strain. The protocol also explores whether program costs are offset by savings in health care utilization and institutionalization compared with usual care.

**Methods:**

In this single-center, controlled trial, 65 patient-caregiver dyads affected by advanced PD (Hoehn and Yahr stages 3-5 and homebound) are recruited to receive quarterly interdisciplinary home visits over 1 year. The 1-year intervention is delivered by a nurse and a research coordinator, who travel to the home, and it is supported by a movement disorder specialist and social worker (both present by video). Each dyad is compared with age-, sex-, and Hoehn and Yahr stage–matched control dyads drawn from US participants in the longitudinal Parkinson’s Outcome Project registry. The primary outcome measure is the change in patient quality of life between baseline and 1 year. Secondary outcome measures include changes in Hoehn and Yahr stage, caregiver strain, self-reported fall frequency, emergency room visits, hospital admissions, and time to institutionalization or death. Intervention costs and changes in health care utilization will be analyzed in a budget impact analysis to explore the potential for model adaptation and dissemination.

**Results:**

The protocol was funded in September 2017 and approved by the Rush Institutional Review Board in October 2017. Recruitment began in May 2018 and closed in November 2019 with 65 patient-caregiver dyads enrolled. All study visits have been completed, and analysis is underway.

**Conclusions:**

To our knowledge, this is the first controlled trial to investigate the effects of interdisciplinary home visits among homebound individuals with advanced PD and their caregivers. This study also establishes a unique cohort of patients from whom we can study the natural course of advanced PD, its treatments, and unmet needs.

**Trial Registration:**

ClinicalTrials.gov NCT03189459; http://clinicaltrials.gov/ct2/show/NCT03189459.

**International Registered Report Identifier (IRRID):**

PRR1-10.2196/31690

## Introduction

### Background

Parkinson disease (PD) is the second most common neurodegenerative condition; however, a substantial proportion of patients with advanced PD are disconnected from clinicians and researchers [1,2]. Many individuals with PD become homebound because of the progressive motor and functional disabilities that their disease imposes. Other comorbidities, limitations, absence of a caregiver, distance from care, or a combination thereof also contribute to a growing number of homebound individuals with PD. Consequently, care becomes fragmented or absent, increasing the likelihood of poor outcomes, including medication errors and other complications [3-6]. Caregivers bear the burden of meeting the needs of these complex and often severely disabled patients. The resulting caregiver strain often leads to institutionalization, excess morbidity, and mortality [7-12].

Little is known about the natural progression of homebound individuals with PD or their caregivers. PD can be staged using the Hoehn and Yahr (HY) scale: HY 1 and 2 comprise mild unilateral and bilateral motor symptoms, respectively; HY 3 signifies moderate symptoms with balance impairment; advanced disease is indicated by HY 4, severe symptoms necessitating an assistive device to walk; or HY 5, which indicates a wheelchair or bedbound status. Our knowledge of advanced PD, treatment strategies, quality of life (QoL), and caregiver outcomes are based primarily on cohorts derived from outpatient clinics. The most advanced and disabled individuals, whose very disease creates tangible barriers to care, are often unable to leave their homes for any variety of necessary clinical visits or research opportunities [13]. An ongoing international observational study is investigating the course of advanced or late-stage parkinsonism [14]; however, this remains limited to individuals accessing outpatient care.

The substantial economic burden of PD has been well described, including a 2017 analysis reporting direct medical costs of US $25.4 billion and an additional US $26.5 billion in indirect costs, including unpaid caregiving time, time spent by the patient and caregivers in contact with services, and lost productivity [15]. However, few economic analyses have been sufficiently powered to examine the costs of care in advanced PD patients, who comprise at most 6%-10% of the largest population-based studies [16]. In a community-based UK PD cohort, direct costs were 184% higher for HY 4-5 patients than HY 1, and indirect costs were 31% higher [17]. In the multinational European Care of Late-Stage Parkinsonism study, in which 93.9% (214/228) of participants were stage HY 4-5, the mean annualized direct care costs were €35,980 (US $42,697.43), which were 166% to 384% higher than previously reported cohorts [16].

In response to other chronic, complex, and disabling conditions of older adults, home visits have re-emerged as a way to maintain continuity of care, avoid institutionalization, and improve QoL [18-21], with equivocal findings on cost-effectiveness depending on the health care system [22-24]. Although the travel, time, and labor costs of home visits exceed traditional outpatient visits, the opportunity to proactively identify previously undetected symptoms, signs, and safety risks may avert crises and acute health care utilization, offsetting or potentially saving costs. The majority of home visit models are interdisciplinary, incorporating primary care, nursing, and social work [19,25-30]. Two specialized home visit programs for PD have been described [31,32]; however, neither used interdisciplinary care, addressed caregiver burden, or defined the population served, the outcomes achieved, or programmatic costs.

In a pilot study, we demonstrated the feasibility of delivering comprehensive, expert, interdisciplinary care via home visits for homebound patients with advanced PD and related disorders [33]. In the initial cohort, 85 individuals with PD or related disorders received 272 home visits over 2 years throughout New York City by a traveling team of a movement disorder specialist, a nurse, and a social worker. Nearly 70% of enrolled patients were rated HY stage 4 or 5 at their first visit (severe symptoms requiring an assistive device to ambulate, or being wheelchair or bedbound, respectively), demonstrating the ability to reach and recruit the target population. Both the program satisfaction and retention exceeded 95%.

To better understand longitudinal changes in homebound individuals, we enrolled a subset of those 85 individuals receiving clinical home visits in a 1-year prospective cohort study. Among the 85, we excluded 58 (68%) individuals for the following reasons: 11 (13%) had atypical parkinsonism, 7 (8%) were non–English-speaking, and 4 (19%) each who died, moved out of the catchment area, expressed no further need for home visits, or declined participation before the enrollment visit, respectively. Finally, we excluded 24 (28%) individuals because of either impaired decisional capacity (Mini-Mental State Examination [MMSE] <10 or nonverbal; 15/85, 18%), or potentially impaired decisional capacity with MMSE <20 and no caregiver to consent (5/85, 6%) or MMSE <20 and caregiver with significant health issues or at risk for caregiver loss (4/85, 5%). However, in the 27 eligible individuals with advanced PD consenting to four visits over 1 year, we found a marked worsening of the Unified Parkinson’s Disease Rating Scale (UPDRS) total score after 1 year, without an accompanying decline in QoL [34]. Although our pilot results suggest that the presumed parallel decline and inextricable connection between PD severity and QoL may be disentangled, the study was limited by size, had restricted geographic diversity, and lacked a control group. To address sustainability and costs, we developed a hybrid approach with the in-home nurse connecting to the physician by video, and the social worker attending initial visits in-home and subsequent visits by video alongside the physician, creating a telehealth-enhanced home visit [35].

### Objective

On the basis of this experience and subsequent modifications as described, we present this protocol for a prospective study of telehealth-enhanced home visits by a movement disorder specialist, a nurse, and a social worker, compared with age-, sex-, and HY stage-matched controls from a national, longitudinal PD registry [36,37]. We hypothesize that providing comprehensive, longitudinal, interdisciplinary, and in-home consultation to individuals with advanced PD and their caregivers might inform and transform care for this growing population. By strategically adding telehealth, many opportunities develop in terms of shared specialty care resources across broader geographical regions [38-40]. We aim to determine the impact of these quarterly home visits on (1) patient QoL, (2) caregiver strain, and (3) caregiver depression and anxiety. As an exploratory aim, we will conduct a budget impact analysis to evaluate the feasibility and cost-effectiveness of this model.

## Methods

### Study Setting and Design

**Figure 1:**
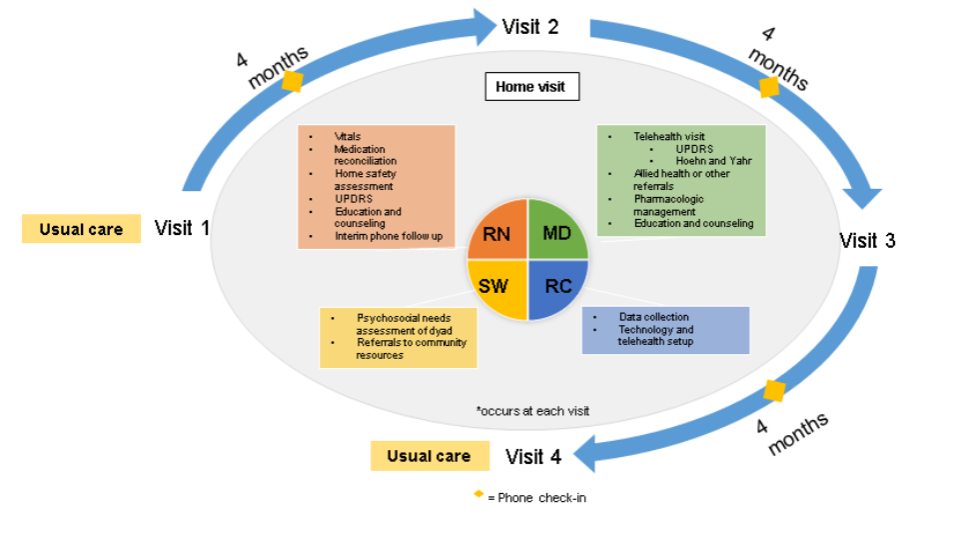
Interdisciplinary home visits for Parkinson disease study structure, visit flow, and discipline-specific responsibilities at each visit. MD: movement disorders specialist; RC: research co-ordinator; RN: nurse; SW: social worker; UPDRS: Unified Parkinson’s Disease Rating Scale.

The Interdisciplinary Home Visits for Parkinson’s Disease (IN-HOME-PD) study is a single-center cohort study of quarterly, interdisciplinary home visits enhanced by telehealth for homebound individuals with advanced PD and their caregivers. Enrolled patient-caregiver dyads are compared with matched controls drawn from the Parkinson’s Foundation (PF) Parkinson’s Outcome Project (POP). Recruitment began on May 7, 2018. IN-HOME-PD participants are recruited from the Rush University Medical Center PF Center of Excellence in Chicago, Illinois. We are enrolling 65 pairs of patients and caregivers. Screenings take place via electronic medical record (EMR) chart review and phone call, whereas visit 1 assessments take place in patients’ homes (Figure 1). Visits 2-4 are performed where the patient resides at the time of the visit (eg, home, skilled nursing facility, or nursing home).

### Patient Inclusion Criteria

The patients must be aged ≥40 years, be seen within the past 2 years at the Rush Movement Disorders Clinic with a diagnosis of PD according to the UK PD Brain Bank criteria [41] from their treating neurologist and HY stage 3-5 at the most recent visit, have one or more criteria for advanced PD as delineated in Textbox 1, and live within 30 miles of the Rush Movement Disorders Clinic. The patients must have a caregiver willing to serve as their study partner, with the respective inclusion and exclusion criteria listed below. Finally, these patients must be considered homebound [42], be community-dwelling (an independent dwelling such as an apartment, condominium, or house owned or rented by, or provided to or shared with the patient), and either demonstrate the capacity to consent, or have a consenting study partner and capacity to assent to participation [43].

**Inclusion and exclusion criteria for Interdisciplinary Home Visits for Parkinson’s Disease patients and caregivers.**

**Interdisciplinary Home Visits for Parkinson’s Disease Patient**

- Inclusion criteria
  Aged ≥40 years
  Diagnosis of idiopathic Parkinson disease by a neurologist
  ≥1 visit in the past 2 years at Rush Outpatient Movement Disorders Clinic
  Hoehn and Yahr stage 3-5 at the most recent clinical visit
  Reside within 30-mile radius of Rush University
  Community-dwelling and homebound
  ≥1 of the following criteria, as determined by the referring neurologist: Motor or cognitive fluctuations Multi-morbidity Medication mismanagement Cognitive impairment Symptoms of depression and/or anxiety High risk for hospitalization or hospital readmission High risk for nursing facility admission Suspected elder abuse Recent history of increased falls at home Suspected caregiver burnout ≥2 canceled or no-show appointments with neurologist in past 12 months
  Caregiver willing to serve as study partner
  Capacity to consent or caregiver consent and assent or caregiver consent without dissent
- Exclusion criteria
  Severe psychiatric disorder interfering with ability to participate in the study, as determined by the referring neurologist or principal investigator
  Non–English-speaking
  Atypical, vascular, or drug-induced parkinsonism
  Subjects without an informal caregiver

**Interdisciplinary Home Visits for Parkinson’s Disease Caregiver**

- Inclusion criteria
  Aged >30 years
  Unpaid individual spending an average of >20 hours weekly engaged in care-related tasks related to the patient-subject
  Capacity to consent
  Agree to participate in nested trial of caregiver peer mentoring
  Working telephone number at which participant can be contacted by study team
- Exclusion criteria
Non–English-speaking
  Active psychosis or other severe psychiatric disease, as reported by participant or determined by study team member during screening
  Terminal illness (life expectancy <12 months)

### Patient Exclusion Criteria

Patients are excluded from participation if they have a severe unstable psychiatric disorder (exclusive of PD psychosis), are non–English-speaking, or have an atypical form of parkinsonism according to the most recent visit with their treating neurologist.

### Caregiver Inclusion Criteria

Caregivers must be aged ≥30 years; demonstrate the capacity to consent; serve as a caregiver to the patient, defined as either cohabitating with the patient or spending an average of >20 hours weekly engaged in unpaid care-related tasks; have a working telephone; and agree to participate in a nested trial of caregiver peer mentoring.

### Caregiver Exclusion Criteria

Caregivers are excluded if they are diagnosed with a severe psychiatric disorder, non–English-speaking, terminally ill (have been told by a medical professional that they have <12 months to live, by self-report), and hired and paid as a formal caregiver in a part-time or full-time capacity.

### Control Participants

The matched control participants are drawn from the POP longitudinal registry [36]. Once all first visits are completed, the team provides the PF with a deidentified data set containing the age, sex, and HY stage of all patients at visit 1. The PF then provides a subset of POP participants with at least two consecutive annual visits and a caregiver study partner, matched to patients by exact HY, sex, and age ±5 years). The pilot feasibility data from the initial New York–based cohort (with similar eligibility requirements to this study) indicated that we could match 93% of those participants on sex, age, and HY stage, to at least two POP controls. Sex, age, and HY stage were selected as matching variables because of their association with PD duration, severity, caregiver strain, and institutionalization [37,44,45]. In the event of insufficient POP matches, the study team will use propensity score matching rather than direct 1:1 matches.

### Recruitment and Screening Strategies

The potential participants are identified and recruited by direct referral from the Rush Movement Disorder Clinic neurologists or via chart review. The study team presents the structure and logistics, eligibility criteria, and referral process to referring neurologists at regular intervals throughout the recruitment period. Referring neurologists contact the coordinator directly with the names of potential participants. The coordinator also prospectively screens clinic schedules and retrospectively queries the EMR for potential participants seen in the past 2 years. If a potential participant is identified in the EMR, the coordinator confirms eligibility with the neurologist before contacting the patient. Once potential participants are provided with additional information about study requirements and confirm interest in the study, visit 1 is scheduled. As of July 2021, recruitment is complete.

### Clinical Assessment

As shown in Table 1, all assessments occur at quarterly visits over a 365-day timeframe (with a 60-day window of flexibility). Visits, interim follow-up calls, and documentation have all been designed to incorporate principles of geriatrics, palliative care, and best practices in the management of PD and to be integrated into the EMR. At visit 1, the nurse, social worker, and coordinator travel to the home and complete the capacity assessment and informed consent process. The coordinator sets up an internet hotspot and tests the tablet connectivity via a Health Insurance Portability and Accountability Act (HIPAA)–secure videoconferencing app. While the coordinator arranges telehealth technology, the nurse assesses the patient for the following: demographics, orthostatic vitals (or supine vitals if bedbound), disease history, and comorbidities.

**Table 1:** Study assessments.

Domains			Instruments		Study visit						
					1^a^		2^b^		3^b^		4^b^
**Patient**											
	Medical history	Standardized initial and interim medical history		✓^c^		✓		✓		✓	
	Orthostatic vital signs	Manual sphygmomanometer		✓		✓		✓		✓	
	Demographics and PD^d^ history	Standardized questionnaire		✓							
	Comorbidities	Self-administered comorbidity questionnaire		✓							
	Medication reconciliation	Standardized questionnaire		✓		✓		✓		✓	
	Nonmotor activities of daily living	UPDRS^e^ I		✓		✓		✓		✓	
	Motor activities of daily living	UPDRS II		✓		✓		✓		✓	
	Physical examination	UPDRS III		✓		✓		✓		✓	
	Motor complications	UPDRS IV		✓		✓		✓		✓	
	PD stage	Hoehn and Yahr		✓		✓		✓		✓	
	Cognitive assessment	Abbreviated MoCA^f^		✓						✓	
	Home safety assessment	Standardized questionnaire		✓							
	Resource utilization questionnaire	Standardized questionnaire		✓		✓		✓		✓	
**Quality of life**											
	Quality of life assessment, long form	PDQ-39^g^		✓						✓	
	Quality of life assessment, short form	PDQ-8^h^				✓		✓			
	Program satisfaction	CSI-SF^i^		✓						✓	
	Satisfaction with telehealth visits	Telehealth satisfaction survey								✓	
**Caregiver**											
	Demographics	Standardized questionnaire		✓							
	Comorbidities	Self-administered comorbidity questionnaire		✓							
	Caregiver strain	MCSI^j^		✓		✓		✓		✓	
	Anxiety and depression	HADS^k^		✓		✓		✓		✓	
	Self-efficacy	Self-efficacy questionnaire		✓		✓		✓		✓	
	Cognition	Abbreviated MoCA		✓						✓	
	Program satisfaction	CSI-SF		✓				✓		✓	
**Postvisit follow-up**											
	Approximately 4 week follow-up phone call	Semistructured template in electronic medical record		✓		✓		✓		✓	

^a^Visit 1: coordinator, nurse, social worker present at home; movement disorder specialist present by video.

^b^Visits 2-4: coordinator and nurse present at home; social worker and movement disorder specialist present in real time via telehealth. Visit 4: 365 (SD 60) days after visit 1.

^c^Domain examined.

^d^PD: Parkinson disease.

^e^UPDRS: Unified Parkinson’s Disease Rating Scale.

^f^MoCA: Montreal Cognitive Assessment (four-item shortened version of MoCA used in the Parkinson’s Outcome Project, including immediate and delayed five-item recall, oral trails, and category fluency).

^g^PDQ-39: Parkinson’s Disease Questionnaire.

^h^PDQ-8: Parkinson’s Disease Questionnaire-Short Form.

^i^CSI-SF: Client Satisfaction Inventory-Short Form.

^j^MCSI: Multidimensional Caregiver Strain Index.

^k^HADS: Hospital Anxiety and Depression Scale.

At each visit, the nurse and dyad complete a medication reconciliation and ensure that the EMR-documented medication list and schedule align with actual administration at home. Specifically, the nurse identifies medication strength and frequency errors, expired medications, and duplicate medications. The nurse also documents errors in omission (ie, not taking prescribed medication) or commission (ie, actively taking a discontinued or deprescribed medication). Any errors detected are relayed to the movement disorder specialist and addressed in the shared assessment and plan.

The nurse performs a standardized checklist-based home safety assessment including the following: safe entrance and exit from the home; fall risks within the home such as unstable throw rugs, poorly lit hallways, or lack of handrails for indoor steps; bathroom and bedroom safety, including bath or shower grab bars, seats, and toilet aids; and presence of working fire alarms, fire extinguishers, and an emergency escape plan.

The team measures both patient and caregiver cognition with a shortened version of the Montreal Cognitive Assessment (MoCA), mirroring the assessment used for POP participants for comparability [36]. Items include immediate and delayed five-item recall, oral trails, and category fluency. To minimize priming or interference with the MoCA, the team engages the caregiver in other assessments while the patient completes the MoCA and vice versa.

The social worker assesses the caregiver’s demographics, comorbidities, strain (Multidimensional Caregiver Strain Index [MCSI]) [46], and mood (Hospital Anxiety and Depression Scale) [47]. The caregiver completes surveys on self-efficacy [48] and satisfaction with preintervention PD care using the Client Satisfaction Inventory-Short Form (CSI-SF) [49]. Finally, the social worker initiates a discussion of goals of care and advance directives with the dyad.

After the nurse and social worker perform their assessments, they call the movement disorder specialist to present their respective data and develop a preliminary plan. While the team members are conferring, the coordinator completes the Parkinson’s Disease Questionnaire (PDQ-39) [50] and the CSI-SF with the patient. For these two assessments, the remaining team members are blinded, and both data collection and entry are completed by the coordinator alone.

Once the nurse, social worker, and movement disorder specialist have conferred, the telehealth component of the visit begins. The movement disorder specialist joins the visit by video (VidyoConnect, Vidyo Inc) and explores and addresses symptoms and concerns. The movement disorder specialist completes a physical examination using observation and prompted actions supported by the nurse, including the Unified Parkinson’s Disease Rating Scale (UPDRS) [51,52]. For rigidity and postural instability items, the registered nurse assesses these items in person with the movement disorder specialist’s supervision. The movement disorder specialist determines the HY score [53]. On the basis of all of the visit assessments, the movement disorder specialist revises and presents a unified plan to address symptoms and unmet needs. Before departure, the team provides an after-visit summary, including an accurate health-literacy–friendly medication schedule and relevant educational material. Each team member completes a templated note in the EMR and later collates it into a comprehensive document shared with all health care providers involved in the patient’s care. Patients are permitted to continue seeing any of their existing health care providers during the course of the study, including primary care providers.

Visits 2-4 (and their corresponding follow-up phone calls) are identical to visit 1, with the following exceptions: the social worker joins via telehealth, and the home safety assessment and static measures, such as demographics, are omitted. At visits 2 and 3, the Parkinson’s Disease Questionnaire-Short Form (PDQ-8) [54], a validated, shortened version of the PDQ-39, is used. At visit 4, the full PDQ-39 is used, and the dyad completes a telehealth satisfaction survey [55]. The duration of the visits, including all clinical and study assessments, is approximately 90-180 minutes.

### Follow-Up Calls

**Figure 2:**
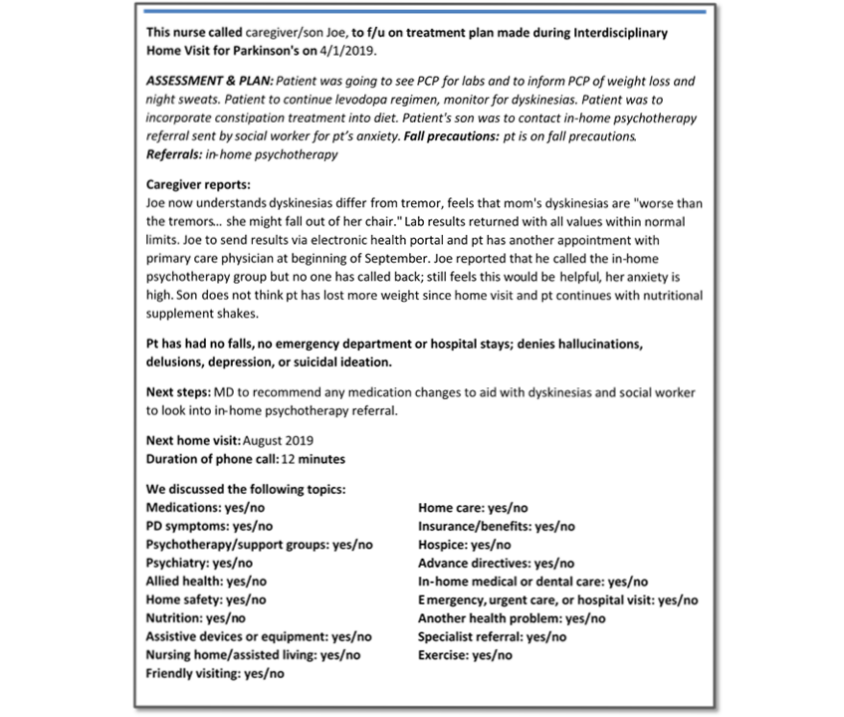
Example of a follow-up phone call note for an interdisciplinary home visit for Parkinson disease in the electronic medical record using a standardized template. MD: movement disorders specialist; PCP: primary care provider; PD: Parkinson disease.

Approximately 4 weeks after each home visit, the team calls the dyad to follow up on the care plan, any updates, and further recommendations from the team or referring neurologist. Again, this is documented in a templated telephone encounter (example in Figure 2) and shared with the relevant team members. Interim calls afford additional opportunities for the detection of clinical deterioration and interdisciplinary case management and intervention.

### Outcome Measures

The instruments illustrated in Table 1 are used to collect data from the patients and their caregivers throughout the study.

#### IN-HOME-PD Patients Only

QoL is assessed using the PDQ-39 (visits 1 and 4) and PDQ-8 (visits 2 and 3). The former is a 39-item, eight-domain tool, with each item scored 0 (never) to 4 (always). The latter is an 8-item version, with each item representing one PDQ-39 domain [50]. Domain scores as well as a summary index score (0-100), can be calculated, with higher scores signifying worse QoL. PDQ-8 is administered at visits 2 and 3 to minimize assessment time and avoid missing data if dyads are lost to follow-up before visit 4. If the patient is unable to complete the PDQ-39 or PDQ-8 because of cognitive impairment, the caregiver may answer on the patient’s behalf.

#### IN-HOME-PD Patients and Caregivers

Both patients and caregivers complete the CSI-SF to measure participants’ satisfaction with the program. The CSI-SF is a nine-item instrument used to assess client satisfaction with multidisciplinary programs. Subjects indicate their satisfaction on a 7-point Likert scale at baseline and at visit 4 [49]*.* At the end of visit 4, dyads complete the telehealth satisfaction survey, a 17-item instrument designed specifically for telemedicine visits in patients with PD [56].

#### Caregivers Only

The MCSI is an 18-item tool measuring six dimensions of subjective responses to stressors in caregivers [46]. Subscales include physical strain, social constraints, financial strain, time constraints, interpersonal strain, and demanding or manipulative stress. Respondents are asked about the frequency with which items apply, ranging from *never* to *all of the time* on a 5-point scale. The 14-item validated Hospital Anxiety and Depression Scale (HADS) measures anxiety and depression [47]. Scores >8 for either anxiety or depression subscores indicate probable symptoms. A nine-item scale measures symptom management self-efficacy [48].

#### Budget Impact Analysis

The exploratory budget impact analysis takes a health care system perspective based on provider time and health care utilization [57]. Budget-related data include prospectively captured research- and intervention-specific costs, telehealth-associated difficulties and delay, item purchases for study use, health care utilization by the patient, and provider time dedicated to their care. We capture health care utilization via a standardized questionnaire from the POP, which includes falls, emergency department visits, hospitalizations, institutionalization, allied health referrals, outpatient care, and binary data on the use of various PD medication categories. The average health care utilization and cost per patient per year will be compared with the use data available through the POP.

The total yearly costs will be divided into intervention and health care utilization costs. The intervention costs are recorded by the study team, who track their program-related activities, costs, and time. Each team member is prompted on a monthly basis to record 1 week’s worth of effort, indicating time spent on study-specific tasks (ie, screening, visit scheduling and preparation, travel, EMR charting, and both scheduled and unscheduled interim calls). All entries are categorized as intervention-related or research-related, and research-related costs will be excluded from the analysis. Health care utilization information will be described for the 12 months before and following baseline for IN-HOME-PD and POP subjects, with descriptive statistics for each category of health care utilization, including emergency department visits, hospitalizations, outpatient services, and use of various PD-related medication categories. In addition, the proportion of subjects institutionalized over the course of 1 year will be included. The total yearly costs (intervention and health care utilization) per IN-HOME-PD patient-year will be calculated and compared with those of POP controls.

### Primary End Point

The primary end point is the change in PDQ-39 over 1 year between IN-HOME-PD patients and controls (visit 1 to visit 4 in IN-HOME-PD patients; annual POP assessments in controls).

### Secondary End Points

Secondary end points include changes in caregiver strain within IN-HOME-PD caregivers over 1 year and between IN-HOME-PD caregivers and matched control caregivers, using the MCSI. Additional secondary patient end points compared between IN-HOME-PD patients and matched controls include self-reported fall frequency, count and presenting complaint for any emergency department visits and hospitalizations, institutionalization, and death. Among the IN-HOME-PD dyads only, we are assessing the change in caregiver anxiety, depression, and self-efficacy, as these variables are not present in the POP database. We are assessing telehealth satisfaction and dyad satisfaction with IN-HOME-PD care, using the change in CSI-SF from visit 1 to visit 4. Finally, exploratory end points include the cost per visit and annualized cost per dyad based on team member time and labor and modeled costs of health care utilization (emergency department visits and hospitalizations) among both IN-HOME-PD patients and controls. Indirect costs, including time spent caregiving or lost wages because of PD or caregiving, are beyond the scope of this study and absent from the POP; thus, we did not include them in this analysis.

### Ethics Approval and Consent to Participate

The Rush University Medical Center Institutional Review Board initially approved this study on October 25, 2017 (current protocol version 8, dated March 19, 2020), and the trial is posted in a national database (registration number NCT03189459). All participants in this study (patients and their caregivers) provided written informed consent. In the event that the patient lacks the capacity to consent because of cognitive impairment, the caregiver may consent on their behalf with the patient giving assent. Participants may withdraw from the study and return to their prior care provider at any time. If a caregiver wants to withdraw but the patient wants to continue participation, a suitable alternative caregiver, approved by the patient’s legally authorized representative, must be willing and able to participate to allow for the patient’s continued participation. If a suitable caregiver is not willing and able to participate, the patient will be withdrawn from the study, and the study team will reconnect the patient with their prior care provider.

### Sample Size

Our preliminary data and recruitment support our ability to enroll 65 dyads in 16 months. On the basis of a 12% attrition rate in our pilot New York–based cohort, we conservatively planned for a 20% drop out rate in this larger study, yielding 52 dyads. This affords 79% power to detect a minimal clinically important between-group difference of 6 in the PDQ-39 summary index [58-60] with a sample size of 50 pairs using an estimated baseline mean of 52.0 (SD 15.0) and α=.05, using a two-sided paired *t* test [61]. This is a conservative estimate given our anticipated higher ratio of matched controls (3-4:1). A sample size of 50 dyads yields 99% power to detect a 10-point difference in change in MCSI (from a mean of 24 to 34, SD 8) over 12 months compared with controls, as measured using a two-sample paired-means test with a significance level of 0.05, based on pilot data. The budget impact analysis is exploratory in nature.

### Data Collection and Monitoring

The team meets weekly, outside of study visits, to identify potential issues with consent or assessment procedures, manage any reported adverse events or unintended effects of home visits, and monitor progress. A REDCap (Research Electronic Data Capture) database was created to house the data, with quarterly audits to ensure fidelity [62]. Data will be exported in deidentified form to Stata 15 (StataCorp LLC) for analysis.

### Statistical Analysis

#### IN-HOME-PD Patients

Demographics and confounders include race, ethnicity, insurance, socioeconomic status (average household income for zip code) [63,64], living situation (home or nursing facility), PD duration (from the year of PD onset or diagnosis, if onset unknown), cognition as measured in the POP using items from the MoCA, and comorbidities (self-reported presence and severity of heart and respiratory problems, diabetes, cancer, arthritis, and other neurological disorders) The following items not in the POP are used in analyses of program dyads only: education, caregiver demographics (age, race, ethnicity, and education), depression, hallucinations, motor severity (UPDRS), satisfaction with the program (CSI-SF), and telehealth satisfaction [56].

Baseline demographics, PD characteristics, and QoL in program and POP subjects, with categorical variables summarized by frequencies and percentages, will be described. Continuous variables will be assessed for normality and summarized as mean and SD or median and IQR, as appropriate. Using chi-square and two-tailed *t* tests, as appropriate, we will evaluate the adequacy of matching [65,66]. We will compare the change in Parkinson’s Disease Questionnaire-Summary Index (PDQ-SI) over 1 year for each matched pair using a paired *t* test or nonparametric Wilcoxon signed-rank test. If a subject dies or is lost to follow-up, the last value of the PDQ-8 SI will be carried forward [54]. We will analyze the association with change in PDQ-SI for each demographic, confounder, and covariate via multivariable analysis of variance to account for matching. We will correct for multiple comparisons as appropriate. We will construct a linear regression model with change in PDQ-SI as the dependent variable and home visits as the primary independent variable. We will use stratified linear regression, accounting for matching variables, to assess the contributions of each as potential confounders. Model building will include manual stepwise backward elimination testing for multicollinearity, confounding, and effect modification. Finally, we will describe patient and caregiver satisfaction with the home visit intervention and telehealth, respectively, analyzing patient and caregiver predictors of change in satisfaction, or predictors of dyadic discordance in satisfaction, if appropriate.

#### Caregivers

We will describe demographics and baseline caregiver strain, anxiety, depression, and satisfaction with their loved one’s preintervention PD care. We will compare within-subject changes in MCSI over 12 months and between-subject changes across IN-HOME-PD and POP caregivers. The last MCSI value will be carried forward in the event of loss to follow-up. We will evaluate the proportion of caregivers in each group with a categorical change in strain (eg, from moderate to severe). We will construct a linear regression model with change in MCSI as the dependent variable, home visits as the primary independent variable, and we will adjust for potential confounders, such as caregiver and patient demographics and cognitive impairment.

#### Budget Impact

We will describe health care utilization in the 12 months before and following baseline, respectively, for program and POP subjects. Health care utilization includes the limited data set assessed in the POP, namely, self-reported frequency of emergency department visits and hospitalizations, along with their admitting diagnoses. In addition, at each home visit, we gather the frequency of primary care, neurologist, and other specialist visits, respectively, and the frequency of phone calls or health portal electronic messages reported by the dyad in the interim since the prior home visit. To calculate the costs of the program (ie, intervention), we will include the study team’s time spent on phone calls, emails, and other intervention-related communication. We will exclude research-related program costs (eg, coordinator time spent on questionnaires not directly pertaining to clinical care) from the budget impact analysis. All program component costs will be summed to calculate total program costs, and program and health care costs will be summed to calculate total costs. We will present descriptive statistics for each category of health care utilization and the proportion of subjects institutionalized over 1 year, defined as a change in living situation from *home* to a *skilled nursing facility or nursing home*. We will compare IN-HOME-PD with POP subjects on hospitalizations, emergency department visits, institutionalization, and total costs during the 1-year observation period using chi-square and *t* tests, as appropriate. We will determine the effect size of the intervention on each use category and analyze the association between each category and each demographic, confounder, and variable of interest via *t* test or analysis of variance.

In the budget impact analysis, multivariable analyses will consist of constructing two model types: (1) multivariable logistic regression models for any hospitalization, emergency department visit, or institutionalization within 1 year (three separate, dichotomous outcomes), and (2) multivariable Poisson regression models for counts of hospitalizations and counts of emergency department visits (two separate count outcomes). More complex models will be built as above, focusing on PD duration, cognitive impairment, QoL, caregiver strain, and prior use as confounders or effect modifiers in the relationship between intervention and use. Using a dependent variable of total costs, we will construct a generalized linear model with log link and gamma distribution, testing for significant differences in total costs by treatment status [67,68]. The achievable savings estimate of implementing this program nationwide will be calculated by extrapolating our results to 5% of the PD population (a conservative estimate of those seen at PF Centers of Excellence) [36]. In sensitivity analyses of the home visit intervention, we will vary the costs of team composition, duration and cost of travel, and geographic region.

## Results

This protocol was funded in September 2017 and approved by the Rush Institutional Review Board in October 2017. Recruitment began in May 2018 and closed in November 2019, with 65 patient-caregiver dyads enrolled and having completed visit 1. When the SARS-CoV-2 pandemic reached the United States and lockdowns went into place in mid-March 2020, all in-person portions of the home visits were converted to video or phone visits on the participants’ own devices, if available, and marked as pandemic-modified visits in the database for subsequent analyses. All dyads enrolled at that time had already completed at least visits 1 and 2. For these pandemic-modified visits, the nurse obtains vital signs gathered on the dyad’s home blood pressure monitoring cuffs, if available, and coaches the caregiver through obtaining orthostatic vital signs. Medication reconciliation is conducted by video or phone with the caregiver, and the movement disorder neurologist conducts a remote UPDRS examination by video whenever possible [52]. Given the advanced stage of many IN-HOME-PD patients and the risk of attrition, the study team determined that it would be preferable to conduct modified quarterly visits on the predetermined schedule rather than defer visits until after the pandemic. In exploratory analyses, we will identify differences in primary and secondary outcomes, overall satisfaction, and telehealth satisfaction among participants completing all visits per protocol and those with pandemic-modified visits.

As of June 2021, all study visits have been completed. Matching is underway, followed by data analysis, with results expected to be published in fall 2021.

## Discussion

### Rationale for This Model

Our prior work in New York City identified an understudied population of advanced homebound PD patients with high symptom and caregiver burden and poor QoL who were amenable to interdisciplinary home visits [33,34]. In pilot studies of home visits, QoL did not significantly decline during the yearlong follow-up, suggesting that expert care delivered directly to the patient-caregiver dyad may mitigate some of the decline previously deemed inevitable. Given the ethical considerations of withholding care from those unable to access it [69,70] and the high dropout rates seen in PD interventions with waitlist controls [70,71], a randomized controlled trial of interdisciplinary home visits is neither appropriate nor feasible. However, matched controls can provide a reasonable comparison group in this understudied population [69].

During the development of the model in 2014-2017, the availability and use of video telehealth increased, with growing interest and evidence to support telehealth as an effective care model in PD [55,72,73]. Particularly in the pre-COVID era, several limitations affected its implementation in the homebound population, including possession of both relevant technology and connectivity, digital literacy [74,75], and neuropsychiatric and sensory impairments that would render unsupported telehealth difficult or impossible, and therefore, reliance on a care partner with the equipment and skills to facilitate telehealth [76,77]. Cognitive interviews with pilot participants revealed a significant amount of paranoia and apprehension regarding new cameras, computers, wires, and other devices being brought into the home. With these concerns in mind, we piloted several telehealth models and determined that the use of a mobile hotspot and tablet brought and operated by the study team, rather than relying on the participants’ own devices or connectivity, was the most efficient and acceptable. Telehealth connectivity and overcoming the digital divide created by users, technology, and internet and cellular barriers will remain an important variable in studying any intervention reliant upon them.

Recruitment is an additional and anticipated challenge inherent to a population that has eluded care and clinical research until recently [14,78-80]. Identifying potentially eligible patients through the EMR offers certain advantages; however, documentation may not reflect the correct diagnosis, stage, or presence of a caregiver. In some instances, the record may not be updated in a timely manner following the patient’s demise; thus, screening phone calls must be handled with sensitivity. In addition, the labor and time intensity of the model required us to determine a catchment area large and geographically diverse enough to meet recruitment goals, however, circumscribed enough to prevent extensive travel time and remain within state boundaries because of licensure limitations.

Despite the challenges of reaching advanced homebound individuals with PD and their caregivers before and during the SARS-CoV-2 pandemic, the potential impact of this and subsequent studies to aid in defining and ultimately addressing QoL and caregiver strain in this population is significant. This is among the first of several studies to longitudinally follow such advanced, underserved patients and caregivers and report on the trajectories of QoL, caregiver strain, and health care utilization. This is also the first study to compare interdisciplinary home visits to usual care for this population and longitudinally investigates both patient, caregiver, and cost outcomes. By standardizing the roles and responsibilities of each team member, including video telehealth, and incorporating templated documentation, this model may be leveraged to foster continuity of care, effective interdisciplinary case management, and improve QoL and caregiver strain for countless homebound individuals with PD and other neurodegenerative diseases.

### Availability of Data and Materials

The deidentified data sets generated and analyzed during this trial will be available from the corresponding author upon reasonable request. Data from all control subjects were retrospective and available to the investigators by request to the PF.

## Appendix

Multimedia Appendix 1 Peer-reviewer report from National Institute of Neurological Disorders and Stroke (National Institutes of Health).

## Abbreviations

CSI-SF: Client Satisfaction Inventory-Short Form
EMR: electronic medical record
HIPAA: Health Insurance Portability and Accountability Act
HY: Hoehn and Yahr
IN-HOME-PD: Interdisciplinary Home Visits for Parkinson’s Disease
MCSI: Multidimensional Caregiver Strain Index
MMSE: Mini-Mental State Examination
MoCA: Montreal Cognitive Assessment
PD: Parkinson disease
PDQ-8: Parkinson’s Disease Questionnaire-Short Form
PDQ-39: Parkinson’s Disease Questionnaire
PDQ-SI: Parkinson’s Disease Questionnaire-Summary Index
PF: Parkinson’s Foundation
POP: Parkinson’s Outcome Project
QoL: quality of life
REDCap: Research Electronic Data Capture
SI: summary index
UPDRS: Unified Parkinson’s Disease Rating Scale
