# Supplementary material for: Longitudinal, Interdisciplinary Home Visits Versus Usual Care for Homebound People With Advanced Parkinson Disease: Protocol for a Controlled Trial
Source: JMIR Res Protoc. 2021 Sep 14;10(9):e31690. doi: 10.2196/31690 (PMC8479607; doi:10.2196/31690)
Supplement: Multimedia Appendix 1 [file resprot_v10i9e31690_app1.pdf]

SUMMARY STATEMENT
(Privileged Communication)

PROGRAM CONTACT:
Beth-Anne Sieber
301-496-5680
sieberb@ninds.nih.gov

Release Date: 03/26/2017
Revised Date:

Application Number: 1 K23 NS097615-01A1

Principal Investigator

FLEISHER, JORI ERIN

Applicant Organization: NEW YORK UNIVERSITY SCHOOL OF MEDICINE

Review Group: NST-1
NST-1 Subcommittee

Meeting Date: 02/27/2017
Council: MAY 2017
Requested Start: 07/01/2017

RFA/PA: PA16-198
PCC: SIEBEBND

Project Title: Reaching the Most Vulnerable: A Novel Model of Care in Advanced Parkinson's Disease
SRG Action: Impact Score:26
Next Steps: Visit https://grants.nih.gov/grants/next_steps.htm
Human Subjects: 30-Human subjects involved - Certified, no SRG concerns
Animal Subjects: 10-No live vertebrate animals involved for competing appl.
Gender: 1A-Both genders, scientifically acceptable
Minority: 1A-Minorities and non-minorities, scientifically acceptable
Children: 3A-No children included, scientifically acceptable
Clinical Research - not NIH-defined Phase III Trial

Project Year	Direct Costs Requested	Estimated Total Cost
1	187,216	202,193
2	187,179	202,153
3	187,017	201,978
4	187,097	202,064
5	186,645	201,576
TOTAL	935,154	1,009,965

ADMINISTRATIVE BUDGET NOTE: The budget shown is the requested budget and has not been adjusted to reflect any recommendations made by reviewers. If an award is planned, the costs will be calculated by Institute grants management staff based on the recommendations outlined below in the COMMITTEE BUDGET RECOMMENDATIONS section.

1K23NS097615-01A1 Fleisher, Jori

RESUME AND SUMMARY OF DISCUSSION: This is the resubmission of a K23 application from Dr. Jori Fleisher who proposes to gain training in clinical research, comparative and cost effectiveness research, palliative care skills research and the implementation and dissemination of science; while identifying, testing, and disseminating effective therapies and models of care for an understudied population of patients with advanced Parkinson's disease (PD). The review panel noted that Dr. Fleisher is a very promising K23 candidate who is an Assistant Professor in the Departments of Neurology and Population Health at NYU. Dr. Fleisher has strong research and clinical training in geriatrics, neurology and movement disorders. She was described as being highly motivated to become an independent researcher in the field of movement disorders. The review panel noted that Dr. Fleisher has an MS in Clinical Epidemiology in addition to her clinical training. Despite two new publications, Dr. Fleisher's publication record is still rather modest. However, the panel noted that Dr. Fleisher has already worked on the development of an interdisciplinary home visit program that was funded by the Edmond J. Safra Foundation. This brought her recognition from several other foundations in the form of funding successes. The panel noted that this proposal features a strong advisory committee, better defined career goals, and a very good gap-based training plan that includes appropriate coursework and meetings with advisors and mentors. The panel noted Dr. Fleisher's mentoring team, all local to NYU, is very strong as it includes Dr. Joshua Chodosh as the primary Mentor and Drs. Alessandro Di Rocco and Heather Gold as co-mentors. The research environment was noted to be outstanding and the institutional support very strong with 80% protected time. The panel noted that between the mentoring team and the advisory committee, all expertise needed for Dr. Fleisher's success in the proposed research is present. One concern regarding, was the ambitious nature of Dr. Fleisher's training plan when it is coupled with the amount of work involved with sitting on several committees and a task force, along with her clinical duties. Given Dr. Fleisher's track record, other panel members were less concerned.

The panel noted that Dr. Fleisher's research focuses upon an area of tremendous interest since it has the potential to create a new model of interdisciplinary home care. More specifically, three aims will determine whether a telemedicine-enhanced home visit program improves quality of life, caregiver strain, and cost-effectiveness for patients with advanced PD. In addition, the proposed research leverages an existing infrastructure that is already led by Dr. Fleisher, and also features a peer mentor intervention to improve quality of life ratings and mood among caregivers. Power calculations were noted to be satisfactory, and appropriate scientific rigor is demonstrated in Dr. Fleisher's methods. The premise was felt to be adequately addressed. If proven effective, this innovative model of in-home care could change clinical practice. Minor concerns included the feasibility of recruitment, the short one-year time period for follow-up testing, and limited assessments in Aims 2 and 3. Regarding the proposed one-year follow-up period, some panel members indicated it may be too short to detect meaningful differences in quality of life, care and mood. These same panel members acknowledged that the concern is minor since it is probably unavoidable due to potential attrition if the time frame between testing is set to longer than one year. Another concern for some was that the preliminary data indicate that the quality of life numbers improve despite evidence of disease progression. Thus, it was unclear if the improved quality of life ratings are clinically meaningful. It also was noted that it was difficult to understand which quality of life numbers were for the patient or for the caregiver. Overall, the review panel had high enthusiasm not only for Dr. Fleisher, but for the proposed research which has potential for high-impact and should prepare Dr. Fleischer to be competitive for future independent funding.

Training in the Responsible Conduct of Research: Acceptable.

DESCRIPTION (provided by applicant): As Parkinson's Disease (PD) progresses, the symptoms of this debilitating and costly condition increase in number and severity, quality of life declines, and

caregiver strain rises, resulting in 100-200,000 US patients becoming homebound. This population loses access to care despite overwhelming need. Evidence supports interdisciplinary and home-based models of care in other elderly cohorts, and the use of telehealth in earlier stages of PD; however, none of these have been formally tested in advanced PD. The long-term goals of this K23 are 1) to develop the candidate into a leader in the fields of movement disorders and health services research, and 2) to develop, test, and disseminate models of care and related interventions to improve access to care, quality of care, and quality of life for patients with advanced PD and their caregivers. The aims of this project are 1) to test the efficacy of an interdisciplinary home visit program for patients with advanced PD on patient quality of life and caregiver strain when compared with usual care; 2) to compare the effects of home visits with and without caregiver peer mentoring on caregiver health; and 3) to conduct a budget impact analysis of the model. We will prospectively study 60 patient-caregiver pairs matched with subjects receiving usual care within the National Parkinson Foundation Parkinson's Outcome Project (POP); we will enroll 36 experienced past caregivers as peer mentors. The K23 candidate is an Assistant Professor of Neurology and Population Health at New York University School of Medicine, and completed a movement disorders fellowship and NINDS T32-supported Master's of Science in Clinical Epidemiology at the University of Pennsylvania. She has conducted clinical and health services research in geriatrics, neurology, and PD, identifying health literacy and medication beliefs as barriers to care. She has demonstrated the feasibility of a pilot interdisciplinary home visit program for homebound individuals with PD. The candidate is committed to a career in patient-oriented research and proposes an comprehensive five-year plan of mentorship, formal training, self-directed learning, and research. She will develop expertise and skills in: 1) Implementation and dissemination science; 2) Comparative and cost effectiveness research; and 3) Palliative care skills and research. The results of this K23 will inform future R01 applications that will test the efficacy and cost effectiveness of this model for advanced PD in other settings, and adapt this model to other chronic neurologic conditions. By identifying, testing, and disseminating effective therapies and models of care for this previously understudied population, we can minimize morbidity and unnecessary healthcare utilization. Dr. Fleisher's mentorship team includes expertise in PD, implementation and dissemination of interdisciplinary care models, telehealth, cost effectiveness, and the POP. The outstanding institutional support, and proposed training, career development, and research plans will ensure Dr. Fleisher's successful transition to an independent clinical investigator dedicated to improving the health outcomes of patients with advanced Parkinson's Disease and other neurodegenerative conditions.

PUBLIC HEALTH RELEVANCE: Advanced Parkinson's Disease is a debilitating, costly, and understudied condition. Improving access to comprehensive, specialized, in-home patient care and caregiver support offers the potential to minimize the downward spiral of morbidity and preventable healthcare utilization. The aim of this proposal is to test whether and to what degree an interdisciplinary home visit program, with and without peer mentoring for caregivers, will improve patient- and caregiver-reported outcomes and reduce healthcare costs when compared with usual care in advanced Parkinson's Disease.

CRITIQUES

NOTE: *The critiques and criterion scores from individual reviewers are provided below in an essentially unedited form. These were prepared prior to the review meeting and may not have been updated or revised subsequent to the discussion at the meeting. Therefore, they may not fully reflect the final opinions of the individual reviewers at the close of group discussion or the final majority opinion of the group. The Resume and Summary of Discussion above summarizes the final outcome of the group discussion.*

CRITIQUE 1
Candidate: 2
Career Development Plan/Career Goals & Objectives: 3
Research Plan: 4
Mentor/Co-Mentor(s), Consultant(s), and Collaborator(s): 2
Environment and Institutional Commitment to the Candidate: 1

Overall Impact: This is a resubmission of a K23 application by a promising candidate with strong research and clinical training in geriatrics and neurology, movement disorders, and Parkinson's disease research. The candidate is highly motivated and has a goal of becoming an independent health services researcher in movement disorders. In addition to her clinical training, she has a MS in clinical epidemiology and has already worked on number of healthcare projects including development of an interdisciplinary home visit program (funded by the Edmond J. Safra Foundation). Her work is now recognized by several other foundations and is garnering increasing interest. Although her publication record remains modest, she has had 2 recent publications of note. Her career goals are well detailed and better identify gaps in training. The coursework outlined appears appropriate, but concern is raised regarding the ambitious nature of her training plan including coursework, committees, task force, and meetings. She has created a solid mentoring team at NYU and outstanding advisory committee that will help ensure her success. Her environment is outstanding and institutional support strong. Her research focuses on an area of tremendous interest in advanced PD and has the potential to create a new model of interdisciplinary home care incorporating a mentoring team and telemedicine. Prior concerns included differentiation of her project, but these have been addressed. Minor concerns include the feasibility of mentor recruitment, time period for follow-up, and limited assessments in Aims 2 and 3. Overall, this project is met with enthusiasm and has potential for high-impact and launching the candidate's career.

1. Candidate:

Strengths

- Highly motivated candidate with strong research and clinical training in geriatrics (Feinberg School of Medicine at Northwestern Univ.) and neurology (U. Penn.), movement disorders, and PD research. She is a current Assistant Prof at NYU.
- Master of Science in Clinical Epidemiology (MSCE) supported by NINDS T32 neuro-epidemiology grant with coursework in biostatistics, research methodology, epidemiology
- Early success: T32 grant support, Samuel Zeritsky Award for Excellence in Research, Interdisciplinary Home Visit Program funded by Edmond J. Safra Philanthropic Foundation; telehealth funding from Parkinson Alliance, NPF, Doris Duke Fund; CurePSP grant for advanced PD/atypical parkinsonism care

Weaknesses

- Modest publication record, but two recent publications added

2. Career Development Plan/Career Goals & Objectives/Plan to Provide Mentoring:

Strengths

- Laudable goal of becoming independent health services researcher in movement disorders. The candidate has the background, training, experience and motivation to help her achieve this goal.
- Short and long-term goals and objectives are well detailed
- Gaps in training identified: training in implementation/dissemination of science (?), palliative care, and comparative effectiveness research. Coursework planned is appropriate.

- Plan for mentorship is solid and includes experts in her field (Primary: Dr. Chodosh) with regular meeting. She has put together an outstanding advisory committee to oversee her progress.

Weaknesses

- Amount of coursework, local and national career development opportunities, committee work, task force, may be overambitious.

3. Research Plan:

Strengths

- The candidate's proposed research focuses on an area of great need and tremendous interest in advanced PD and has the potential to forward an innovative model of (in-home) care, and change practice, for many patients with PD. The goals of her project fit well with her background, expertise, and long-term interests.
- Based on successful pilot Interdisciplinary Home Visit Program
- Candidate addressed gaps in her approach to advanced PD: targeting caregiver strain, added peer mentoring, and telemedicine.
- Rigor is demonstrated and power calculations included.

Weaknesses

- 1-year follow-up period may be too short to detect meaningful differences in quality of care and impact of patients, caregivers, and mood. This concern may be minor and unavoidable due to attrition and confound of increase in comorbid conditions.
- Aims 2 and 3 rely on one outcome measure: MCSI, and HADS, respectively.
- Preliminary data and feasibility of mentor recruitment and capability is lacking.

4. Mentor(s), Co-Mentor(s), Consultant(s), Collaborator(s):

Strengths

- Primary mentor, Dr. Chodosh, though relatively new to NYU is outstanding. He has expertise in the candidate's field of interest, is well-funded, and has a proven track record of successfully mentoring. Strong letter.
- Co-mentors, Drs. Di Rocco and Gold, are excellent and provide expertise in advanced PD care, health disparities, dissemination of health care technologies, and cost-effectiveness analyses.
- Strong advisory committee including Dr. Mengling Liu (NYU), Peter Schmidt (NPF), and Jayne Wilkinson (U Penn).

Weaknesses

- Dr. Chodosh has not mentored a K-awardee before, but this is a minor weakness as he has trained many other junior faculty.

5. Environment and Institutional Commitment to the Candidate:

Strengths

- The candidate is in an outstanding environment that fosters collaboration and support.
- Strong support expressed by her Chair. 80% protected time stipulated, start-up funds, department resources, and support for her mentor team.

Weaknesses

- None significant

Training in the Responsible Conduct of Research: Acceptable

Comments on Format (Required):

- formal course, lectures, discussion groups

Comments on Subject Matter (Required):

- covers all 9 recommended topics

Comments on Faculty Participation (Required):

- unclear

Comments on Duration (Required):

- adequate

Comments on Frequency (Required):

- adequate, refresher courses included

Authentication of Key Biological and/or Chemical Resources: Not Applicable (No Relevant Resources)

Protections for Human Subjects: Acceptable Risks and Adequate Protections

- there is minimal risk in this study, questionnaires and clinical information collected and stored

Inclusion of Women, Minorities and Children: Acceptable

- Sex/Gender: G1A: Both sexes used, Scientifically acceptable
- Race/Ethnicity: M1A: Minority and non-minority, Scientifically acceptable
- For NIH-Defined Phase III trials, Plans for valid design and analysis: Not applicable
- Inclusion/Exclusion of Children under 18: C3A: Adults Only (>18 yr.), Scientifically Justified

Vertebrate Animals: Not Applicable (No Vertebrate Animals)

Biohazards: Not Applicable (No Biohazards)

Resubmission: Acceptable

- The candidate has adequately addressed reviewer comments

Select Agents: Not Applicable (No Select Agents)

Resource Sharing Plans: Acceptable

Budget and Period of Support: Recommend as Requested

CRITIQUE 2
Candidate: 3
Career Development Plan/Career Goals & Objectives: 3
Research Plan: 5
Mentor/Co-Mentor(s), Consultant(s), and Collaborator(s): 1

Environment and Institutional Commitment to the Candidate: 1

Overall Impact: This is revised application from a neurologist with specialty training in movement disorders fellowship in 2014. The revised application is modestly responsive to the previous critiques. A concern raised previously was the extent of experimental publications, which remains limited. The training plan has been revised to focus on implementation science and dissemination of outcomes, supported by short courses. The research plan addresses an important area, but the project has some methodological challenges related to the implementation of the peer-caregiver intervention. Overall this is a responsive application that addresses an important area. The project will be more challenging than perhaps recognized, but the outcomes will facilitate the candidate's career.

1. Candidate:

Strengths

- The candidate received an MD in 2008, completed residency in neurology in 2012, and fellowship in movement disorders in 2014.
- The candidate completed a MSCE in clinical epidemiology, pharmacoepidemiology in 2015.

Weaknesses

- Experimental publications remain thin.

2. Career Development Plan/Career Goals & Objectives/Plan to Provide Mentoring:

Strengths

- The career development plan has been revised to highlight implementation science and dissemination research, comparative and cost analysis research, implementation of science, and comparison of health outcomes.
- Comparative effectiveness research training program (NYU) Palliative Care Education and Practice Program (Harvard), and Implementation Science Certificate Program (UCSF).
- The PCEP includes a 7 day intensive learning course at Harvard followed by a 6 month weekly online course discussion.

Weaknesses

- The time requirement to complete the online training (e.g., Implementation Science) is significant. The applicant may wish to reduce the total burden by selecting only those courses most germane to the training goals.

3. Research Plan:

Strengths

- The aims will test 3 hypotheses to determine whether a telemedicine-enhanced home visit program for advanced PD improves quality of life, caregiver strain, and cost-effectiveness.
- The research will leverage an ongoing infrastructure already led by the applicant.
- A peer mentor intervention is proposed to improve QOL ratings and mood among caregivers.

Weaknesses

- Preliminary data indicate that patient QOL improved despite progression of "severe PD disability". However, the raw data are not provided, and the duration period is not provided. It is unclear if the improved QOL ratings are significantly and clinically meaningful.

- Difficult to understand when patient QOL and caregiver QOL is the focus in the preliminary data.
- Despite the updated interest in implementation science, the rigor of this approach in the preliminary data is limited.

4. Mentor(s), Co-Mentor(s), Consultant(s), Collaborator(s):

Strengths

- The mentorship team is strong.

Weaknesses

- No significant concerns.

5. Environment and Institutional Commitment to the Candidate:

Strengths

- The environment is strong.

Weaknesses

- No significant concerns.

Training in the Responsible Conduct of Research: Acceptable

Comments on Format (Required):

- acceptable

Comments on Subject Matter (Required):

- acceptable

Comments on Faculty Participation (Required):

- acceptable

Comments on Duration (Required):

- acceptable

Comments on Frequency (Required):

- acceptable

Authentication of Key Biological and/or Chemical Resources: Acceptable

Protections for Human Subjects: Acceptable Risks and Adequate Protections

- acceptable

Inclusion of Women, Minorities and Children: Acceptable

- Sex/Gender: G1A: Both sexes used, Scientifically acceptable
- Race/Ethnicity: M1A: Minority and non-minority, Scientifically acceptable
- For NIH-Defined Phase III trials, Plans for valid design and analysis: Not applicable
- Inclusion/Exclusion of Children under 18: C3A: Adults Only (>18 yr.), Scientifically Justified

Vertebrate Animals: Not Applicable (No Vertebrate Animals)

Biohazards: Not Applicable (No Biohazards)

Resubmission: Acceptable

Select Agents: Not Applicable (No Select Agents)

Resource Sharing Plans: Acceptable

Budget and Period of Support: Recommend as Requested

CRITIQUE 3
Candidate: 2
Career Development Plan/Career Goals & Objectives: 2
Research Plan: 2
Mentor/Co-Mentor(s), Consultant(s), and Collaborator(s): 2
Environment and Institutional Commitment to the Candidate: 1

Overall Impact: Revised submission. Candidate dealt with the prior reviewers and detailed them well. Well trained candidate with good support. This novel program can impact care and provide a foundation for future funding. Some concerns about the caregivers as mentors but this is negligible. Career development plan is based on gaps.

1. Candidate:

Strengths

- Strong training and support

Weaknesses

- *Reviewer did not provide any comments under weaknesses*

2. Career Development Plan/Career Goals & Objectives/Plan to Provide Mentoring:

Strengths

- Gap based.

Weaknesses

- *Reviewer did not provide any comments under weaknesses*

3. Research Plan:

Strengths

- Figures describe the flow. Has continued to collect preliminary data Good statistical plans.

Weaknesses

- *Reviewer did not provide any comments under weaknesses*

4. Mentor(s), Co-Mentor(s), Consultant(s), Collaborator(s):

Strengths

- Strong mentors

Weaknesses

- *Reviewer did not provide any comments under weaknesses*

5. Environment and Institutional Commitment to the Candidate:

Strengths

- Strong chair letter with startup and protected time of 80%.

Weaknesses

- *Reviewer did not provide any comments under weaknesses*

THE FOLLOWING RESUME SECTIONS WERE PREPARED BY THE SCIENTIFIC REVIEW ADMINISTRATOR TO SUMMARIZE THE OUTCOME OF DISCUSSIONS OF THE REVIEW COMMITTEE ON THE FOLLOWING ISSUES:

PROTECTION OF HUMAN SUBJECTS (Resume): ACCEPTABLE.

SEX/GENDER: G1A: BOTH SEXES USED, SCIENTIFICALLY ACCEPTABLE

RACE/ETHNICITY: M1A: MINORITY AND NON-MINORITY, SCIENTIFICALLY ACCEPTABLE

INCLUSION/EXCLUSION OF CHILDREN UNDER 18: C3A: ADULTS ONLY (>18 YR), SCIENTIFICALLY JUSTIFIED

VERTEBRATE ANIMAL (Resume): NOT APPLICABLE.

BIOHAZARD COMMENT: NONE.

MODEL ORGANISM SHARING PLAN: NOT APPLICABLE.

DATA/RESOURCE SHARING: ACCEPTABLE.

COMMITTEE BUDGET RECOMMENDATIONS: The budget was recommended as requested.

Footnotes for 1 K23 NS097615-01A1; PI Name: Fleisher, Jori Erin

NIH has modified its policy regarding the receipt of resubmissions (amended applications). See Guide Notice NOT-OD-14-074 at http://grants.nih.gov/grants/guide/notice-files/NOT-OD-14-074.html. The impact/priority score is calculated after discussion of an application by averaging the overall scores (1-9) given by all voting reviewers on the committee and multiplying by 10. The criterion scores are submitted prior to the meeting by the individual reviewers assigned to an application, and are not discussed specifically at the review meeting or calculated into the overall impact score. Some applications also receive a percentile

ranking. For details on the review process, see http://grants.nih.gov/grants/peer_review_process.htm#scoring.

MEETING ROSTER
NST-1 Subcommittee
Neurological Sciences Training Initial Review Group
NATIONAL INSTITUTE OF NEUROLOGICAL DISORDERS AND STROKE

NST-1
02/27/2017 - 02/28/2017

Notice of NIH Policy to All Applicants: Meeting rosters are provided for information purposes only. Applicant investigators and institutional officials must not communicate directly with study section members about an application before or after the review. All questions should be directed to the Scientific Review Officer in charge of the study section. Failure to observe this policy will create a serious breach of integrity in the peer review process, and may lead to actions outlined in NOT-OD-14-073 at https://grants.nih.gov/grants/guide/notice-files/NOT-OD-14-073.html and NOT-OD-15-106 at https://grants.nih.gov/grants/guide/notice-files/NOT-OD-15-106.html, including removal of the application from immediate review.

CHAIRPERSON(S)

ROSAND, JONATHAN, MD
PROFESSOR
DEPARTMENT OF NEUROLOGY
MASSACHUSETTS GENERAL HOSPITAL
HARVARD MEDICAL SCHOOL
BOSTON, MA 02114

ACTING CHAIR

RICHERSON, GEORGE B, PHD, MD
PROFESSOR AND CHAIRMAN
DEPARTMENT OF NEUROLOGY
THE UNIVERSITY OF IOWA
IOWA CITY, IA 52242

MEMBERS

ADEOYE, OPEOLU M, MD *
ASSOCIATE PROFESSOR
DEPARTMENT OF EMERGENCY MEDICINE
AND NEUROSURGERY
COLLEGE OF MEDICINE
UNIVERSITY OF CINCINNATI
CINCINNATI, OH 45219

AOUIZERAT, BRADLEY E, PHD *
PROFESSOR AND DEPUTY DIRECTOR
BLUESTONE CENTER FOR CLINICAL RESEARCH
DEPARTMENT OF ORAL AND MAXILLOFACIAL SURGERY
NEW YORK UNIVERSITY
NEW YORK, NY 10010

BAAS, PETER W, PHD *
PROFESSOR
DEPARTMENT OF NEUROBIOLOGY & ANATOMY
DREXEL UNIVERSITY COLLEGE OF MEDICINE
PHILADELPHIA, PA 19129

BIEBER, ALLAN JAMES, PHD *
ASSOCIATE PROFESSOR
DEPARTMENT OF NEUROSURGERY AND NEUROLOGY
MAYO CLINIC ROCHESTER
ROCHESTER, MN 55905

BRASHEAR, ALLISON, MD
PROFESSOR AND CHAIR
WAKE FOREST SCHOOL OF MEDICINE
DEPARTMENT OF NEUROLOGY
WINSTON SALEM, NC 27157

BRUCE-KELLER, ANNADORA J, PHD
ASSOCIATE PROFESSOR
DEPARTMENT OF INFLAMMATION
AND NEURODEGENERATION
PENNINGTON BIOMEDICAL RESEARCH CENTER
LOUISIANA STATE UNIVERSITY
BATON ROUGE, LA 70808

CAMPANA, WENDY M., PHD *
PROFESSOR
DEPARTMENT OF ANESTHESIOLOGY
PROGRAM IN NEUROSCIENCE
UNIVERSITY OF CALIFORNIA, SAN DIEGO
LA JOLLA, CA 92093

CAMPBELL, CLAUDIA MICHELLE, PHD *
ASSOCIATE PROFESSOR
DEPARTMENT OF PSYCHIATRY AND BEHAVIORAL
SCIENCES
JOHNS HOPKINS UNIVERSITY
BALTIMORE, MD 21224

CHANG, ANNE-MARIE, PHD *
ASSISTANT PROFESSOR
DEPARTMENT OF BIOBEHAVIORAL HEALTH
PENNSYLVANIA STATE UNIVERSITY
STATE COLLEGE, PA 16802

DOUGHERTY, PATRICK M, PHD *
PROFESSOR
DEPARTMENTS OF PAIN MEDICINE & ANESTHESIOLOGY
MD ANDERSON CANCER CENTER
UNIVERSITY OF TEXAS
HOUSTON, TX 77030

FRIEDMAN, BENJAMIN WOLKIN, MD *
ASSOCIATE PROFESSOR
ALBERT EINSTEIN COLLEGE OF MEDICINE
MONTEFIORE MEDICAL CENTER
DEPARTMENT OF EMERGENCY MEDICINE
BRONX, NY 10467

GALLO, GIANLUCA, PHD *
ASSOCIATE PROFESSOR
DEPT. OF ANATOMY & CELL BIOLOGY SCHOOL OF MEDICINE
TEMPLE UNIVERSITY
PHILADELPHIA, PA 19140

GENDELMAN, HOWARD E, MD *
MARGARET R LARSON PROFESSOR AND CHAIR
DEPARTMENT OF PHARMACOLOGY
AND EXPERIMENTAL NEUROSCIENCE
COLLEGE OF MEDICINE
UNIVERSITY OF NEBRASKA MEDICAL CENTER
OMAHA, NE 68198

GERHARDT, GREG A., PHD
PROFESSOR & SCIENTIFIC DIRECTOR
DEPT. OF NEUROSCIENCE, NEUROLOGY & NEUROSURGERY
UNIVERSITY OF KENTUCKY MEDICAL CENTER
LEXINGTON, KY 40536-0298

GHILARDI, M. FELICE MARINA, MD
PROFESSOR
DEPARTMENT OF PHYSIOLOGY, PHARMACOLOGY AND NEUROSCIENCE
CUNY MEDICAL SCHOOL
NEW YORK, NY 10031

GREGORY, SIMON G, PHD *
PROFESSOR
DEPARTMENTS OF MEDICINE, NEUROLOGY, & MOLECULAR GENETICS AND MICROBIOLOGY
DUKE MOLECULAR PHYSIOLOGY INSTITUTE
DUKE UNIVERSITY MEDICAL CENTER
DURHAM, NC 27710

HARRIS, ANDREW L, PHD *
PROFESSOR
DEPARTMENT OF PHARMACOLOGY, PHYSIOLOGY
AND NEUROSCIENCE
RUTGERS NEW JERSEY MEDICAL SCHOOL
NEWARK, NJ 07103

HECKMAN, BERNADETTE DAVANTES, PHD *
ASSOCIATE PROFESSOR
DEPARTMENT OF PSYCHOLOGY
OHIO UNIVERSITY
ATHENS, OH 45701

LHATOO, SAMDEN, MD, MBBS *
PROFESSOR
DEPARTMENT OF NEUROLOGY
CASE WESTERN RESERVE UNIVERSITY
CLEVELAND, OH 44106-5040

LUCAS, TIMOTHY H, MD, PHD *
ASSISTANT PROFESSOR
DEPARTMENT OF NEUROSURGERY
CENTER FOR NEUROENGINEERING & THERAPEUTICS
UNIVERSITY OF PENNSYLVANIA
PHILADELPHIA, PA 19104

MACMILLAN, CAROL, MD *
ASSOCIATE PROFESSOR
DEPARTMENT OF PEDIATRICS
UNIVERSITY OF CHICAGO
CHICAGO, IL 60637

MANFREDI, GIOVANNI, PHD, MD *
PROFESSOR
DEPARTMENT OF NEUROLOGY AND NEUROSCIENCE
BRAIN AND MIND RESEARCH INSTITUTE
WEILL MEDICAL COLLEGE OF CORNELL UNIVERSITY
NEW YORK, NY 10065

MARSH, ERIC D, MD, PHD *
ASSISTANT PROFESSOR
DIVISION OF CHILD NEUROLOGY
CHILDREN'S HOSPITAL OF PHILADELPHIA
PHILADELPHIA, PA 19104

MCFARLAND, NIKOLAUS RENZ, MD, PHD *
ASSISTANT PROFESSOR
DEPARTMENT OF NEUROLOGY
UNIVERSITY OF FLORIDA
GAINESVILLE, FL 32610

MEINZER, CAITLYN N, PHD *
ASSISTANT PROFESSOR
DEPARTMENT OF PUBLIC HEALTH SCIENCES
MEDICAL UNIVERSITY OF SOUTH CAROLINA
CHARLESTON, SC 29425

MIAO, HONGYU, PHD *
ASSOCIATE PROFESSOR
DEPARTMENT OF BIOSTATISTICS
SCHOOL OF PUBLIC HEALTH
UNIVERSITY OF TEXAS HEALTH SCIENCE CENTER, HOUSTON
HOUSTON, TX 77030

O'BRYANT, SID E, PHD
PROFESSOR
INSTITUTE FOR HEALTHY AGING
UNIVERSITY OF NORTH TEXAS
FORT WORTH, TX 76107

OJEMANN, JEFFREY G, MD
PROFESSOR AND VICE CHAIR
DEPARTMENT OF NEUROLOGICAL SURGERY
UNIVERSITY OF WASHINGTON
SEATTLE, WA 98195

PANIGRAHY, ASHOK, MD *
PROFESSOR
CHILDREN'S HOSPITAL OF PITTSBURGH OF UPMC
CLINICAL AND TRANSLATIONAL IMAGING RESEARCH
DEPARTMENT OF RADIOLOGY
UNIVERSITY OF PITTSBURGH MEDICAL CENTER
PITTSBURGH, PA 15224

PARENT, JACK M, MD
PROFESSOR
DEPARTMENT OF NEUROLOGY
UNIVERSITY OF MICHIGAN SCHOOL OF MEDICINE
ANN ARBOR, MI 48109

PAUL, ROBERT H, PHD *
PROFESSOR
DEPARTMENT OF PSYCHOLOGY
UNIVERSITY OF MISSOURI, ST LOUIS
ST LOUIS, MO 63121

PETERS, JURRIAAN M., MD
ASSISTANT PROFESSOR
DE-ARTMENT OF NEUROLOGY
BOSTON CHILDREN'S HOSPITAL
HARVARD MEDICAL SCHOOL
BOSTON, MA 02115

POWERS, SCOTT W, PHD *
PROFESSOR
DEPARTMENT OF PEDIATRICS
UNIVERSITY OF CINCINNATI
DIRECTOR, BEHAV. SCI. CORE & CLIN. AND TRANS. RES.
CINCINNATI CHILDREN'S HOSPITAL
CINCINNATI, OH 45229

QUINONES-HINOJOSA, ALFREDO, MD
PROFESSOR AND CHAIR
DEPARTMENT OF NEUROLOGIC SURGERY
MAYO CLINIC COLLEGE OF MEDICINE
JACKSONVILLE, FL 32224

SHAH, LUBDHA, MD
ASSOCIATE PROFESSOR
DEPARTMENT OF RADIOLOGY
UNIVERSITY OF UTAH HEALTH SCIENCES CENTER
SALT LAKE CITY, UT 84132--214

SHOYKHET, MICHAEL, PHD, MD *
ASSISTANT PROFESSOR
DEPARTMENT OF PEDIATRICS
WASHINGTON UNIVERSITY
ST LOUIS, MO 63110

TANSEY, MARIALOURDES G, PHD
ASSOCIATE PROFESSOR
DEPARTMENT OF PHYSIOLOGY
EMORY UNIVERSITY SCHOOL OF MEDICINE
ATLANTA, GA 30322

TIWARI-WOODRUFF, SEEMA K., PHD *
ASSOCIATE PROFESSOR
DIVISION OF BIOMEDICAL SCIENCES
SCHOOL OF MEDICINE
UNIVERSITY OF CALIFORNIA, RIVERSIDE
RIVERSIDE, CA 92521

WILEY, RONALD GORDON, PHD, MD
PROFESSOR
VA TVHS
DEPARTMENT OF NEUROLOGY & PHARMACOLOGY
VANDERBILT UNIVERSITY
NASHVILLE, TN 37212

WISNIEWSKI, THOMAS M, MD
PROFESSOR
DEPARTMENTS OF NEUROLOGY,
PSYCHIATRY AND PATHOLOGY
NEW YORK UNIVERSITY SCHOOL OF MEDICINE
NEW YORK, NY 10016

YOSHOR, DANIEL, MD *
PROFESSOR AND CHAIR
DEPARTMENT OF NEUROSURGERY
BAYLOR COLLEGE OF MEDICINE
HOUSTON, TX 77030

SCIENTIFIC REVIEW OFFICER

BENZING, WILLIAM C., PHD
SCIENTIFIC REVIEW OFFICER
SCIENTIFIC REVIEW BRANCH
DIVISION OF EXTRAMURAL RESEARCH
NATIONAL INSTITUTE OF NEUROLOGICAL DISORDERS
AND STROKE, NIH
BETHESDA, MD 20892

EXTRAMURAL SUPPORT ASSISTANT

HOLLEY, SHAVONNA L.
EXTRAMURAL SUPPORT ASSISTANT
SCIENTIFIC REVIEW BRANCH
DIVISION OF EXTRAMURAL RESEARCH
NINDS/NIH/HHS
BETHESDA, MD 20892

MOXLEY, TANYA
EXTRAMURAL SUPPORT ASSISTANT
SCIENTIFIC REVIEW BRANCH
DIVISION OF EXTRAMURAL RESEARCH
NINDS/NIH/HHS
BETHESDA, MD 20892

* Temporary Member. For grant applications, temporary members may participate in the entire meeting or may review only selected applications as needed.

Consultants are required to absent themselves from the room during the review of any application if their presence would constitute or appear to constitute a conflict of interest.
